# Artificial intelligence in neuro-oncology: Current advances and legal considerations in the European Union

**DOI:** 10.1093/noajnl/vdag083

**Published:** 2026-04-03

**Authors:** Sarina Agkatsev, Michael Templeton, Lazaros Lazaridis, Hartmuth Nowak, Sied Kebir, Corinna Seliger

**Affiliations:** Department of Neurology, University Hospital Knapps­chaftskrankenhaus Bochum, Ruhr University Bochum, Bochum, Germany; Assessor of Law, Ludwig-Maximilians-Universität München, München, Germany; Department of Neurology, University Hospital Knapps­chaftskrankenhaus Bochum, Ruhr University Bochum, Bochum, Germany; Center for Artificial Intelligence, Medical Informatics and Data Science, University Hospital Knappschaftskrankenhaus Bochum, Ruhr University Bochum, Bochum, Germany; Department of Neurology and Center for Translational Neuro- and Behavioral Sciences (C-TNBS), Division of Clinical Neuro-oncology, University Medicine Essen, University Duisburg-Essen, Essen, Germany; Department of Neurology, University Hospital Knapps­chaftskrankenhaus Bochum, Ruhr University Bochum, Bochum, Germany

## Abstract

Applications utilizing artificial intelligence (AI) have become a ubiquitous, indispensable part of modern life. This also applies to medicine where AI systems can support staff in decision-making processes and administrative tasks, but there is still uncertainty about the legality of their use. This review introduces examples from neuro-oncology, highlighting legal aspects and providing suggestions for the application of the Medical Device Regulation, the European Regulation on Artificial Intelligence (AI Act), the General Data Protection Regulation, and potential liability issues. AI systems for medical use are medical devices and high-risk AI systems that require the necessary certifications and entail strict requirements for clinical use. Since this may also apply to general AI tools if they are used for medical purposes other than their intended use cases, such use must be critically examined. However, the ultimate responsibility for using AI systems in a medical setting lies with the physicians, who need to be appropriately trained and made aware of the rules and regulations.

Key Points

- Artificial intelligence (AI) can support with decision making and administrative tasks.
- AI systems must comply with the Medical Device Regulation (MDR), AI Act, and GDPR.
- Healthcare professionals must consider liability and exercise due diligence.

Artificial intelligence (AI) is already being successfully utilized in many different areas of life, including medicine. More and more scenarios are conceivable in which AI can improve patient care and increase efficiency of the medical work force; everyday administrative practices in the medical field could also be rendered more structured and practical with the help of AI.

AI is often understood to mean systems that imitate human thought and behavior. It is frequently regarded primarily as systems that are based on machine learning (ML) methods (Figure 1).^1^ ML, or supervised ML, is a branch of AI that is based on mathematical methods that enable a computer to recognize patterns in existing data in an autonomous way, and create a model based on this.^2^ Commonly used ML methods are logistic multivariable regression, support vector machines, or decision tree-based methods (eg random forest). In addition, there are also artificial neural networks, which represent a separate sub-entity of ML. A neural network is a computational model inspired by the structure and function of biological neural systems, consisting of interconnected artificial neurons (nodes) organized into layers. These networks process input data by propagating information through weighted connections, transforming it into outputs via non-linear activation functions. Deep learning (DL) is a subfield of machine learning that employs neural networks with multiple hidden layers (deep architectures) to model complex, hierarchical representations of data, enabling the extraction of intricate patterns and features from large datasets (Figure 1). ML and DL methods could be utilized in neuro-oncology along various points in the patient care pathway. They can be used to help with the patient’s diagnosis,^3^^,^^4^ support the interpretation of magnetic resonance imaging (MRI) scans^5^ or analysis of tissue samples^6^; they also show potential for predicting patient prognosis based on imaging features.^7^ These are merely a few examples how AI could significantly support physicians working in neuro-oncology. In this review we have focused on examples from neuroradiology and neuropathology.

**Figure 1.**
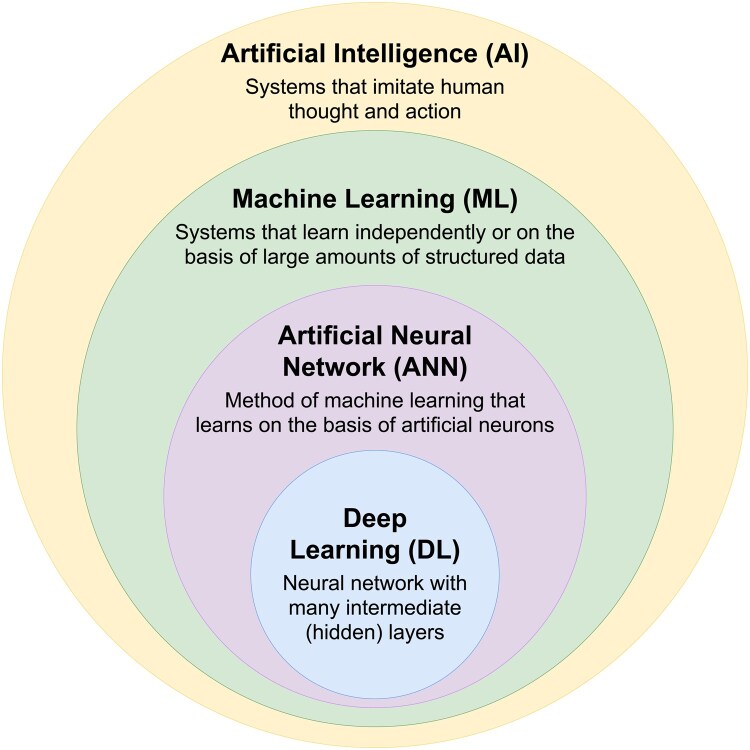
Graphical representation of the nomenclature relating to artificial intelligence. AI = artificial intelligence; ANN = artificial neural network; DL = deep learning; ML = machine learning.

The challenges in training ML models initially lie in the availability and quality of data.^8^ Data preprocessing plays a critical role in addressing these challenges, involving multiple steps to clean, transform, and organize data to ensure its quality meets the requirements for effective model training. High-quality input data is essential for achieving robust and reliable model performance. Once these requirements have been met, the selection of the ML method and the variables to be included in the model (the so-called input variables) are of crucial importance. This information is complemented with the events or values that the model is to predict (output). However, the performance of a trained model is often limited despite its high data quality and quantity. For example, models that have been trained predominantly on data from young, healthy male patients, for instance, are not necessarily transferable to other patient cohorts. This phenomenon is known as “bias.”^9^ Once a ML model overcomes this barrier and works well under different conditions, it is said to be generalizable.

Despite some great advances in the use of AI, there is still uncertainty about the rules and regulations when using AI in medicine and medical administration. In this narrative review, we have therefore highlighted some legal aspects to consider when using AI in these areas, specifically focusing on current developments and future possibilities for AI use in neuro-oncology. The goal of this review is to raise awareness, provide advice, and offer guidelines to stakeholders, such as developers, users and deployers, for using AI applications in medical practice.

This article will therefore examine the Medical Device Regulation (MDR)^10^ and the European Regulation on Artificial Intelligence (AI Act)^11^ for approval and use, as well as important issues for the application of AI in the medical field. Additionally, we will outline, discuss, and offer suggestions regarding the application of the General Data Protection Regulation (GDPR).^12^ Finally, some liability issues regarding the use of AI in practice are briefly addressed.

It is, however, important to bear in mind that the legal framework around the use of AI is currently being adapted in the European Union (EU). Hence, this narrative review is meant to be taken as a snapshot of what is currently possible with AI and what might be possible in the future.

## Search Strategy and Selection Criteria

For this review, different types of searches were conducted:

- In order to illustrate the experimental use of AI in neuro-oncology, the database PubMed was searc­hed using combinations of the terms “artificial ­intelligence,” “neuro-oncology,” “glioma,” “glioblastoma,” “neuropathology,” and “neuroradiology.” Meaningful and recently published articles illustrating examples of AI use in neuro-oncology, in particular neuropathology and neuroradiology, were included. Additional relevant articles, referenced within these papers, were also identified.
- To illustrate examples of AI use in administrative areas and real-life settings PubMed was searched using combinations of the terms “artificial intelligence,” “AI,” “ChatGPT,” “large language models,” “neuro-oncology,” “medicine,” “documentation,” and “clinical documentation.” Additionally, in order to identify AI use in real-life settings a wider search on the search engine Google was performed. Further pertinent articles were identified from papers resulting from the referenced lists of searches.
- To identify relevant literature for the legal part of this review, legislation and documents, commentaries on legislation, books, and articles at national and European level have been searched. The following search terms were used: “medical device regulation,” “MDR,” “General Data Protection Regulation,” “GDPR,” “AI Act,” “AI in medical field,” “AI as a medical device,” “medical liability.” Publications in German and English were reviewed. The final references were compiled based on relevance to the new legislation.

## AI Use in Patient Care in Neuro-Oncology

### Examples of AI Use: Decision Support for Physicians

There are different areas in which AI can support physicians working in neuro-oncology, ranging from neurosurgery, neuroradiology, radiation oncology to neuro- and molecular pathology.^13^ AI could help physicians to establish a brain tumor diagnosis, estimate the prognosis of brain tumors, and help with the therapeutic management.^13^

In neuroradiology, AI algorithms are being investigated for their use in analyzing MRI data and can help to determine tumor volume, type, and size.^14^ The process of extracting information from MRI scans to use for generating quantitative imaging biomarkers is applied in the field of radiomics, and generally consists of three steps: segmentation,^15^ feature extraction, and predictive modeling using ML methods.^16^ Furthermore, it has been demonstrated that by using artificial neural networks (ANN) it is possible to perform an automated calculation of the time to progression by analyzing the tumor volume ­dynamics.^17^ While the above-mentioned examples describe AI use in experimental settings, there are also medical devices that use AI algorithms and serve as decision support in clinical practice. One such example is mdbrain, an AI-assisted software used in neuroradiology, which helps to distinguish and analyze different brain tumor types using a 3D convolutional neural network with U-Net architecture for volumetric segmentation applied on MRI scans.^18^ Another application that is currently used in neuroradiological clinical practice in the EU is icobrain, an AI software assisting physicians with longitudinal brain MRI monitoring,^19^ although the brain tumor-specific module (icobrain tumor) is currently meant to be used for research purposes only. The image analysis software AI-Rad Companion Brain MR provides another example of how automatic segmentation and quantitative analysis of brain structures can support clinicians with interpreting MRI scans.^20^ While this software is also not stated to be used specifically for brain tumor analyses, it is conceivable that in the future there will be a similar application for brain tumor-specific use.

ML algorithms can also be used to determine the presence of molecular markers based on MRI data and thus determine the grade of central nervous system (CNS) tumors according to the World Health Organization (WHO). If automated determination of the WHO grade before surgery were done routinely, it would simplify the diagnosis of tumors, especially in patients with inoperable tumors or if the amount of available tumor tissue were insufficient for histopathological analysis.^21^ It is conceivable that it may even speed up the decision as to whether or not patients are eligible to take part in a clinical trial.^22^

Another area in which AI algorithms can be used in neuroradiology is to differentiate pseudoprogression from real tumor progression,^23^ or to differentiate between various CNS diseases, such as multiple sclerosis and WHO grade 2-4 gliomas.^24^

In neuropathology, trained AI applications are able to distinguish high-grade from low-grade gliomas using digitalized histopathology images of gliomas.^25^^,^^26^ Other studies have shown that it is possible to differentiate between individual brain tumor entities and grades.^27^ Efforts have also been focused on predicting the presence of certain markers and thus extrapolate the molecular classification of the brain tumor entity.^3^ To our knowledge, in neuropathology, AI algorithms are still mainly experimental, and have not been adopted in clinical practice, yet.

On the other hand, interestingly, AI applications are already routinely used in radiation oncology. Software, such as ART-Plan^28^ or AI-Rad Companion Organs RT^29^ use AI algorithms to assist physicians in segmenting and contouring anatomical regions in the head or contouring organs at risk, respectively, in patient images.

### Legal Assessment/Evaluation

Before artificial intelligence can be used in patient care, such as in the above-mentioned examples, and not just for research purposes, numerous legal issues need to be addressed. In doing so, not only the current legal status but also future legislation must be considered, as some recently enacted laws are not yet fully effective. In addition, the regulations will further be refined in the future by legal evolution in the form of further special legislation or jurisdiction.

**Figure 2.**
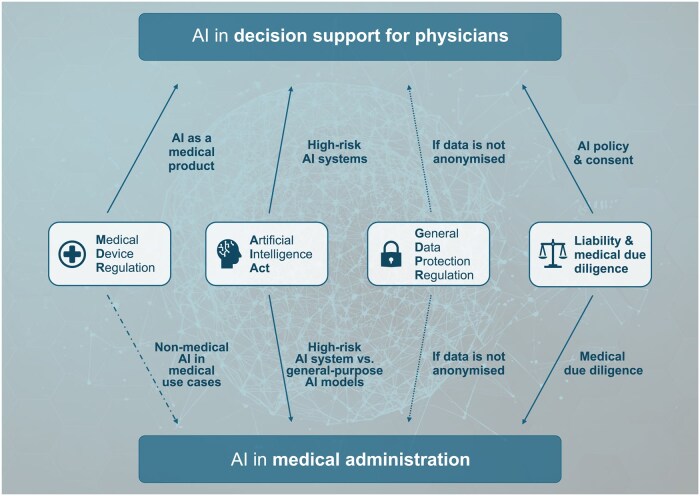
Overview of different rules and regulations that AI systems, used as decision support for physicians and in medical administration, have to comply with. AI = artificial intelligence.

The following sections will discuss the four key legal areas for the use of AI in medicine and the specific issues they raise (Figure 2).

#### AI as Software According to the MDR

The crucial regulation for the manufacturing, distribution, and use of medical devices is the European MDR (p. 43).^10^^,^^30^

AI systems for medical purposes, as well as AI software that is integrated into a medical device, are generally classified as a medical device in the sense of the MDR in Art. 2 para. 1 in conjunction with the guidelines of the Medical Device Coordination Group (MDCG).^10^^,^^31^^,^^32^ Depending on the intended purpose and the associated risks, a classification is made into different risk classes (p. 351).^33^ Rule 11 of the Annex to the MDR classifies software, and thus also AI, in risk class IIa if it is used for diagnostic or therapeutic purposes. If serious deterioration of health or surgical intervention may follow, the classification is increased to class IIb, and in the case of the possibility of irreversible deterioration of health or death to class III (p. 354).^33^

Almost all commercial medical devices on the European market that use AI that find their use in the field of radiology fall into risk class IIa or higher.^34^ For these devices, a conformity assessment procedure at a state-designated body is required by the manufacturer in accordance with Art. 52 et seq. of the MDR before deployment.^35^ The products are then provided with a Conformité Européenne (CE) marking in accordance with Art. 20 MDR, which can be verified through the manufacturer.

In the case of self-learning, dynamic AIs, new conformity assessments may be necessary at regular intervals due to their constant self-adaptation and further self-development.^36^

Thus, when using AI in medical systems, care should be taken to ensure that the necessary certifications are in place. In addition, it is essential that AI systems, comparable to other medical devices, are subject to constant control and review.

#### AI in the Healthcare Sector as a High-Risk System According to the AI Regulation

In August 2024, the European Regulation on Artificial Intelligence (AI Act) came into legal force.^11^ It aims to promote the responsible development and use of AI in the EU. The provisions of the AI Act will only develop their full effectiveness over the next few years, with provisions for high-risk AI systems taking full effect by August 2027.^11^

Hereafter, systems that fall under the regulation of other European provisions and must undergo a conformity assessment, will be classified as high-risk systems following Art. 6 AI Act. In addition to this assessment, there are also instructional, quality management, and reporting obligations (p. 475).^37^ The same applies when the AI system is the medical device itself or part of a safety component in a medical device. Thus, these medical devices that are intended to be used in clinical practice need to be certified according to both the MDR and the AI Act (p. 249).^33^

AI systems that are put into service for the sole purpose of scientific research and development are exempt according to Art. 2 No. 6 of the AI Act. The same exemption applies according to Art. 2 No. 8 AI Act to research and development activities, as long as they do not feature testing in real world conditions.

Art. 26 of the AI Act stipulates multiple obligations for users of such high-risk systems, mainly but not exclusively:

- When selecting such a system, care should be taken to ensure that it meets the necessary requirements, such as the above-mentioned CE marking on conformity according to Art. 48 AI Act, and that the system is registered in the EU’s database of high-risk systems, Art. 49 AI Act (p. 567),^38^ as well as complying with the other provisions of the AI Act (p. 624).^39^ As these provisions have not been fully implemented or set up, yet, such as the EU database for high-risk AI systems, the manufacturers have not had to put these rules into practice yet.
- When operating high-risk systems, appropriate technical and organizational measures must be taken to ensure that the system is used in accordance with the instructions for use. In addition, according to Art. 14 AI Act, the operation requires human oversight and must be carried out by a natural person with the necessary AI literacy as stated in Art. 4 and Art. 3 No. 56 of the AI Act, that is, the training and authority to implement and monitor the system (p. 237).^40^ This must include technical knowledge, experience, education, and training to use the AI system proficiently, taking into account the respective rights and obligations under the AI Act. In addition, these individuals must be aware of the opportunities and risks of AI and the potential damage that it can cause (p. 225).^40^
- In addition, mechanisms for record-keeping (Art. 12 AI Act) must be installed to store the logs generated (automatically) by the system and to immediately inform the system provider and the responsible market surveillance authority in the event of a serious incident (Art. 73 AI Act) within the scope of Art. 3 No. 49 AI Act, that is, serious personal injury or property damage, or the violation of obligations under Union law intended to protect fundamental rights (p. 237).^40^
- When using the system, care must also be taken to ensure clean data entry to guarantee the best possible functioning of the system. Furthermore, the operator has a special duty of transparency. They must inform the natural persons concerned that they are subject to the use of a high-risk system (p. 564).^38^
- Bodies that are governed by public law or private entities providing public service, such as healthcare, must also conduct a fundamental rights impact assessment (FRIA) (p. 579).^38^ This is to ensure that state actors and authorities comply with fundamental rights to privacy, autonomy, data protection, freedom of expression, equality, and health.^41^ In addition, this assessment can be combined with the required data protection impact assessment (DPIA), in order to assess the consequences of the planned data processing operations with regard to the protection of personal data according to Art. 26 para. 9 AI Act and Art. 35 para. 1 GDPR.^12^

Even though these requirements are not mandatory until August 2027 for systems that came into the market or have been significantly altered after August 2026 (Art. 111 No. 2 AI Act), they should be prepared for in the best possible way to ensure the legally compliant future use of AI in the medical sector.

#### Data Protection Assessment in Accordance With the GDPR

Both the MDR and the AI Act refer to the provisions of the European GDPR.^12^ Data protection issues arise in the development and in the use of medical products with AI. Under the GDPR, the EU regulates the handling of personal data. An emphasis is placed on physical and genetic characteristics to underline that the protection of personal data, especially in the context of technological development, is taken into full account (p. 240).^42^

In Art. 2 para 1, the application of the GDPR requires the processing of personal data. A simple way to comply with the regulation would therefore be to anonymize the data used as mentioned in Recital 26 of the GDPR. Data that does not relate to an identified or identifiable natural person or that has been anonymized in such a way that the person in question can no longer be identified, is no longer considered personal data and therefore falls outside the scope of the GDPR (p. 40).^43^ In this context, factual anonymization is sufficient if it is not expected that the data can be re-identified without disproportionate time and effort (p. 106).^44^

The precise way in which anonymization is necessary in practice, especially for use in machine learning systems, depends on the exact type of data used.^45^ Since this anonymized use of data is particularly important for scientific purposes, some data protection authorities are already providing initial guidelines for this.

In principle, it should be possible to use anonymized data for training AI, since personal references do not appear necessary for the general training of AI. However, special cases could arise if the personal data must be part of the data input. In this case, pseudonymization can facilitate the requirements for processing personal data, especially around “big data” as mentioned in Recital 28 of the GDPR (p. 56).^43^

Data from deceased individuals, on the other hand, is unproblematic because the GDPR in Recital 27 does not recognize any postmortem protection of personal rights (p. 254),^46^ as deceased persons can no longer exercise them (p. 86).^44^

In the context of medical treatment and research, the GDPR refers in Art. 9 para. 1 to the data used as data concerning health. This includes all data that contains information about the past, present, or future physical or mental health of the person concerned as clarified in Recital 5 of the GDPR. There may also be overlaps with biometric and genetic data, which are generally suitable for uniquely identifying a person (p. 89).^43^ Although MRI scans of the brain are not considered genetic or biometric data, they are at least considered data concerning health under the GDPR, since a trained observer can draw conclusions about the health status of the person depicted.^47^

Data concerning health, as well as biometric and genetic data, are particularly sensitive data within the meaning of Art. 9 para. 1 of the GDPR. Processing of this data is therefore generally prohibited and only possible under strict regulations (p. 397).^46^

The primary basis for permission is hereby consent, as regulated in Art. 9 para. 2 lit. a. and Art. 6 para. 1 GDPR, which must be given voluntarily and consciously. This can prove difficult in the case of a lack of physical or mental capacity. However, consent can be waived to protect vital interests, but this should only be used as a last resort if the data processing cannot be based on any other legal basis. Other permitted justifications include processing for the purposes of health care, research, and statistics (p. 127).^48^

Alternatively, the GDPR contains another equivalent permission in Art. 9 para. 2 lit. j and Art. 89 for scientific or historical research purposes. This facilitates the processing of personal data insofar as the guarantees and interests of the data subjects listed in Art. 89 GDPR are sufficiently ensured (p. 288).^49^

It should also be noted that special requirements apply to data transfers to third countries, i.e. non-European countries that cannot ensure the required level of protection of the GDPR (p. 892).^50^

Thus, the European legislator has already taken the first steps not only to regulate the use of AI, but also to promote it and keep Europe globally competitive.

#### Liability Issues When Using AI

Lastly, liability issues in the context of the use of AI-supported systems in the medical field, both in diagnosis and therapy, will be outlined briefly.

The use of AI in clinical practice adds further aspects to the classic risk areas of iatrogenic damage. Physicians are in principle free in their choice of method (“therapeutic freedom”). This also applies to the use of technical systems. However, they must comply with a certain medical standard (“specialist standard”) (p. 316),^51^ but such standards have not yet been developed for the use of AI. Thus, according to current working practices, it is necessary to focus on objective due diligence.^52^

However, the use of new methods (“uncharted methods”) is necessary for medical development (p. 476).^53^ This must be done with consideration of the risks, especially due to a lack of experience, as well as the possible chances of recovery. In addition, the patient must be informed in detail about the corresponding risks, in particular the use of the unknown method (p. 478).^53^^,^^54^

Together with the monitoring obligation prescribed by the AI Act, this results in further difficulties. Due to the lack of transparency underlying AI (“black box problem”), it is nearly impossible to understand the AI’s result and thus to recognize errors in its decision-making.^52^ The attending physician, however, is not subject to any warranty liability for technical device errors,^55^ but only for violations of due diligence.^56^ However, these can also consist of using such a system without the necessary care or understanding. The physician must therefore familiarize themselves with the system to the extent that an assessment of safe use can be made (p. 478).^53^

In October 2024, the EU adopted the new Directive on liability for defective products.^57^ This now explicitly includes software as a product. In addition, developers, manufacturers, and providers of AI systems, as defined by the AI Act, are considered to be manufacturers within the meaning of Recital 13 of the Directive.

The fully harmonized Directive grants natural persons the right to compensation in the event of defective software. To facilitate the enforcement of claims, the Directive offers some evidence relief, such as a reversal of the burden of proof and the disclosure of evidence, especially in cases of technical or scientific complexity.^58^

Since this is not a regulation but a directive, it must first be incorporated into national law by the member states. However, due to full harmonization, the level of protection provided cannot be fundamentally deviated from.^58^ The period for implementation runs until December 2026 and should only apply to products that are placed on the market or put into service after that date.

This regulation is intended to ensure that individuals, and thus primarily consumers, are protected from the harm caused by defective products. However, it does not release operators of such products from any duty of care. Even if the systems are not defective, there are numerous due diligence obligations under the above-mentioned provisions that could result in liability on the part of the operator or the physician providing treatment. Therefore, it should be ascertained prior to use whether the existing liability insurance, in particular professional indemnity insurance, covers the remaining risks.

## AI Use in Medical Administration

### Examples of AI Use: Medical Administration

Embedding AI solutions in administrative tasks in neuro-oncology could lead to optimized workflows and thus make the everyday lives of medical staff more efficient. One example is the use of large language models (LLMs), such as the chatbot ChatGPT. The use of chatbots is imaginable in everyday medical practice, for example, when it comes to shared decision-making in neuro-oncology, involving patients and physicians in the treatment decisions as well as improving patient education and follow-up care.^59^ This could lead to increased patient engagement and a more supportive patient-physician relationship.^59^ Interestingly, a study conducted on real health questions and answers in an online forum has already demonstrated that ChatGPT provides more informative and empathetic answers to patient questions than real physicians.^60^ This would support the suggestion of using LLMs as a type of interactive information service and is particularly important (in neuro-oncology) as it is well known that the patients’ trust in the physician plays an important role in treatment, patient satisfaction, and patients’ quality of life.^61^ Furthermore, ChatGPT could also be used as a tool to help with the diagnosis of brain tumors, as demonstrated in a study that used ChatGPT’s ability to diagnose examples of neuro-oncology cases of brain tumors and provide treatment plans.^62^

Another area where LLMs could support, is the induction or knowledge transfer of medical content for doctors or medical students (eg as a ‘virtual tutor’).^63^ It is also possible that AI programs could provide administrative assistance for clinicians and thereby improve clinical documentation. An example of such an application is Dragon^®^ Medical One, an AI-assisted speech recognition software used in dictation devices in neuro-oncological practice for automatic medical speech recognition and simultaneous transcription. It is also feasible that conversations between physicians and patients could be automatically recorded and documented with the help of AI-supported programs.^64^ LLMs have also been investigated for their use in clinical documentation, e.g. for clinical text summarization.^65–67^ Another approach to speeding up the documentation of medical records would be the integration of AI programs for the (semi-)automated creation of medical reports. It would be conceivable for AI programs to combine relevant information from various sources (eg medical reports, diagnostic findings, tumor board decisions) in order to create new documents based on this information.^68^ Since clinical neuro-oncology is burdened with extensive documentation, all of the above-mentioned ways to ease the workload on medical staff would be welcome.

### Legal Assessment of General AI Tools

When using general-purpose AI tools as mentioned in Art. 3 No. 63 of the AI Act, the decisive factor is whether these tools are intended for medical purposes and thus are to be classified as medical devices within the meaning of the MDR. The manufacturer’s intended purpose is generally key here (p. 69).^30^ General AI tools do not automatically become a medical device even if they are suitable for medical use in general or are used for this purpose by a third party.^69^ This also applies to tools like ChatGPT, even if it can provide answers to medical questions.^70^

The crucial aspect is that there is a medical purpose that goes beyond a general one, such as storing data or translating text. This is particularly the case if the AI derives specific diagnoses or therapy recommendations from the used data, or independently creates medical reports from patient and treatment data, which then serve as the basis for further treatment.^70^ In this case, a classification into the corresponding risk classes of the MDR and AI Act is made according to the criteria mentioned above, and if the AI product does not have such a classification and the associated certification, the use for medical purposes cannot be recommended. This seems sensible given that these general-purpose AI tools were not designed for a medical purpose and the associated risk to the legal interests at stake is way too high. Such tasks should only be performed by an AI that was specifically developed and certified for these medical purposes. General AI tools like ChatGPT have recently changed their usage policies to reflect these restrictions and prevent provision of tailored advice that requires a license, such as legal or medical advice, without appropriate involvement by a licensed professional.^71^

When using general tools in clinical practice for non-medical purposes or under the supervision of trained medical personnel, the boundaries are different. If the tools have no impact on medical decisions and are therefore not considered to be medical devices within the meaning of the MDR, a complex certification procedure is ruled out. However, these systems could still be classified as high-risk systems within the meaning of the AI Act if they meet the relevant requirements. The conditions would then be comparable to those mentioned above. The EU Commission has provided corresponding guidelines with examples for the classification into high-risk and non-high-risk systems.^72^

A system can currently be classified as non-high risk if the software merely improves decisions that have already been made or performs preparatory tasks and thus does not pose a significant risk to the health, safety, or fundamental rights of natural persons (p. 311).^73^

In some circumstances, the requirements of Art. 50 AI Act for low-risk systems may still apply. This primarily concerns systems that communicate with people and generate artificial audio, image, or video content. In these cases, the artificial nature of the content must be explicitly stated (p. 761).^74^

AI systems that do not fall into these risk classes are not subject to any special regulations and can be used freely as long as the user has sufficient AI literacy (see above) and possible restrictions concerning personal data are considered (p. 235).^40^ With regard to liability, it can be assumed that a check of the data produced by the AI by a medically trained person should satisfy the duty of care.^75^

Therefore, AI tools can be used in medical administration. However, the individual tool should be examined closely to determine which requirements it must meet for use in everyday clinical practice.

## Conclusion

The use of AI in patient care in neuro-oncology, as well as in clinical administration is affected by numerous regulations and its use poses certain risks and raises questions regarding the patient-physician relationship, the potential loss of knowledge and experience on the part of the medical community. The high requirements for its use and administrative hurdles for its deployment, also regarding data privacy, require an immense personal and financial commitment. AI can also present liability issues in the event of treatment errors, as well as pose challenges with the continuous validation of the training data sets over the entire period of use of the algorithms. In addition, the lack of transparency in the decision-making process (“black box problem”) of AI systems still harbors unforeseen dangers. In these complex systems, strange errors can occur. These systems must therefore be closely monitored, and the results need to be checked thoroughly. It is certain that the ultimate responsibility for the use of medical AI applications must always lie with the medical professionals. The use of AI in patient care in neuro-oncology and in medical administration, therefore, requires careful consideration of all requirements and risks, especially for AI systems that are developed outside the EU and thus outside the scope of the European regulations of the MDR, AI Act, and the GDPR.

However, European legislators have already reacted to recent developments and issued numerous regulations intended to enable the use of AI as a medical device as well as general AI in a secure environment. It is to be expected that the current uncertainties, which arise in the early days, should be resolved after a brief period, as has been the case with other Europe-wide regulations such as the GDPR. Then, existing templates and guidelines can be used to allow much easier practical implementation of these AI systems.

The use of AI systems offers numerous opportunities. The processing of “big data” becomes harder each day and classical technological advancements cannot keep up with the immense benefits and efficiency that AI-featured products offer even today. The advantages that AI-assisted tools can provide in the field of neuro-oncology, as well as in general medical administration are enormous and anyone who misses out on these innovations can expect to face a competitive disadvantage in the future. As development continues, it is highly likely that AI in the medical field will become standard practice, making its use unavoidable.

The legal simplifications for research and testing allow easy entry into the use of AI and can help in getting the framework ready for a future use in clinical practice.
